# Mechanical Dentistry

**Published:** 1873-08

**Authors:** J. F. Canine


### MECHANICAL DENTISTRY.

BY J. F. CANINE, D. D. S.

GENTLEMEN :—The subject of Mechanical Dentistry having been assigned me, I will on the following pages present a few thoughts on the subject. As the object of these meetings is to elevate our profession, let us not lose sight of any one branch while attending to the advancement of another. Therefore, I deem it proper at the outset to say, that mechanical dentistry, in the dental associations of the present day, has been ignored,or at least it has been treated with so little consideration that it has almost been lost sight of, notwithstanding a large proportion of the dentists are occupied the greater part of their time in laboratories practicing this branch of the profession. I have no doubt but every dentist here to-day will corroborate the statement, when I assert that our greatest difficulties are found in mechanical dentistry. Then why treat it so lightly? Is the problem too hard to solve, or is it because we are afraid we might be styled *mechanical* instead of *operative* dentists? I hope not—for it is my candid conviction there are fewer men in our profession to-day masters of this department than of any other branch of the profession.

As I have attempted to write upon this subject, I will commence at the first step in making artificial dentures. I will merely allude to the preparation of the mouth, and pass, briefly as I can, over the several stages necessary to complete the process. The extraction of the teeth I do not care to discuss. But I wish to make a suggestion in regard to trimming down all the prominent parts of the alveolar process with excising or cutting forceps, allowing the gum to close over and heal smoothly. This should be done at the same sitting that the teeth are extracted. It will also be found that there will not be near as much shrinkage. In cases of temporary sets, where this is properly done, the teeth are worn with much more satisfaction than where nature is left to do its own work.

The first step after the preparation of the mouth is taking the impression. Full or upper sets I take in Plaster Paris. There are many failures in this particular of the work, from a lack of knowledge of the proper manipulation. Judgment and tact are indispensable in producing the most desirable results, a perfect impression. To get this, select a cup well adapted to the mouth, soften a piece of wax, (if an upper impression), and place on the posterior edge of the cup, and across from one condyle to the other, place the cup in the mouth and press it up to its place, then remove and trim the wax, leaving but a very narrow surface to come in contact with the posterior platine surface of the mouth. It should extend a little beyond where the plate should go, though not so far as to gag the patient. The wax is placed there to prevent the plaster from sliding back on to the soft parts, and annoying the patient. In preparing the plaster pour sufficient water to mix the requisite amount in a cup, adding a little salt to quicken the setting. Plaster is added until it becomes a thin batter, when it should be well beaten with a spatula until it is smooth and receives a tough appearance.

It is now ready for placing in the impression cup, and should be carefully watched until the proper moment for inserting it in the mouth, when it should be firmly pressed to its proper place, and allowed to remain until the plaster is well set. The time requisite is from one to two minutes.

The lower impression is taken in the same way, only leaving out the wax. In partial sets I use wax first, and frequently nothing else. If I wish to use plaster I cut away the wax where the pla_e is to be, and fill the impression thus prepared with plaster as previously described. Place it in the mouth, being careful to get it in its proper position and up to its place. After the plaster is set the impression is removed, and we are ready to make the model.

To prevent the cast from adhering to the impression use soap and water made into a suds. After the impression is thoroughly saturated with this, wipe off the scum from it with a mop of cotton. Casts made in this way will readily separate by rapping on the cup and cast alternately, unless under cuts prevent.

For the articulatiou arrange wax on plates fitted to the casts. Trim the wax as you imagine the teeth should be. This is in articulating full sets. The wax should be allowed to become hard. Each plate should be placed in the mouth separately, and trimmed until the muscles are relieved and the plate remains in place. This is of great importance, to allow freedom to the lips that they may assume a natural appearance, by which the length and fulness of the teeth may be determined. I cannot impress too strongly the necessity of a thorough knowledge of this part of the work.

It is the cause of all our troubles, or at least many of them. This knowledge is woefully deficient. The results of ignorance in this particular intrudes upon our eyes in ghastly specimens of artificial teeth wherever we go, on the street, at public gatherings; or if you like take a broader field, and travel East, West, North or South, and you meet the same revolting spectacle.

Then why are we not trying to overcome these deficiencies?

Full well I know what an imperfect articulation has cost me. More trouble and annoyance is experienced from this one cause than from all others in constructing artificial dentures. Hence the importance of carefully adjusting this portion of our work accurately, as not only time and labor will be saved, but the accomplishment of the desired result fully and perfectly obtained.

After the trial plates are properly prepared, the wax on them, and trimmed, place the lower piece in the mouth, heat the proximate surface of the antagonizing upper wax, place it in position, and direct the patient to close the mouth ; the hard wax will make its impression on the soft ; the upper plate is removed and trimmed where it is necessary, warmed again on the surface, and replaced in position ; this is continued until the bearings are level, and the jaws are the proper distance apart ; require the patient then to open his mouth wide, and direct him to *drop* his back teeth together. I use this phrase in order to decoy my patient to a proper occlusion of the jaws instead of a false one, which is commonly the case. By directing the patient's attention to the back teeth we avoid the projection of the under jaw, and when you tell the patient to *drop* the back teeth together, he is apt to do so without thought, thereby obtaining the natural position, just what we want. With the jaws properly closed, draw three lines perpendicularly in the wax, right, front and left, and carefully remove from the mouth. In articulating a full upper set, place a small block of wood on the trial plate, place it in the mouth, and trim the wood until it allows the jaws to stand the proper distance apart ; put softened wax on the ridge and over the wood, and direct, as before described, to close the back teeth, press the wax down well around the lower teeth, then remove to the articulation. In partial sets, from one to six teeth, it is my common practice to get the *bite*, at the same time I take the impression. I do this, by simply warming wax and placing it in the vacant spaces where teeth are to be inserted, requiring the mouth to be closed tightly, while the wax is pressed around the teeth. When the wax is placed upon the model the plaster should be trimmed around the teeth to allow the wax to go to its place perfectly. In using gold, or other swaged plates, the wax should be arranged on the plates, and the articulation obtained in the way described, both for partial or full dentures.

The trimming and preparation of the plaster model is of more importance than it would at first seem to most practitioners. Doubtless a vast difference will occur to some of you, between making a close fitting plate on a model, and a close fitting plate in the mouth. You are all aware that a cast has the same unyielding density over its entire surface, while the mouth and particularly the palated portion, has hard and soft tissues, yielding and unyielding in their nature. In order to remedy these differences it is necessary to scrape away portions of the plaster model to correspond with the soft parts in the mouth, trim according to the case, if the roof is flat. trim but little, if soft and spongy, trim the part of the cast that represents that part of the mouth. Trim all models in proportion to their width and depth. Deep wide mouths should be trimmed more than shallow or flat ones, trim from the centre of the alveolar ridge to the palatal surface, until you reaeh the roof or hard parts. After the plate is made it frequently becomes necessary to relieve that part of the plate resting on the condyles, in order that it may go up to its place in the roof. We find others that rock in the mouth, and seem to ride in the roof. We should always be able to see what, and where, the dfficulty is, in order to remedy it without injuring the work. Many plates are damaged beyond redemption by attempting to make them fit, without knowing what the matter is. This I think occurs most frequently in partial sets; some instances in my practice I can recall, where I have defaced their beauty and utility, by grinding and trimming where it was not needed.

Do not trim or deepen your plaster models by guess ; but shape the trial plate to the mouth, in the palatine surface and elsewhere, remove and place it on the model, and trim it until the plate fits. In snagged plates this practice will be found most desirable.

Concerning metallic dies, and swaging plates, I will offer a few suggestions which may not be familiar to all present. I mould in fine sand, using a flask in two sections, and taking but a little more than I wish the plate to cover. With a camels hair pencil I apply fine soap stone or French chalk; put on top upper section of the flask, and dry the mould well, it is now ready to receive the metal, of which I make the die, place the flask on an anvil, or some iron block *after* the zinc is melted, then pour the metal and set the melting cup or ladle on top of the flask. The cold anvil chills the face of the die, while the hot metal in the cup or ladle keeps the top in a melted condition, until the face or the bottom of the cast is thoroughly hardened, causing the largest shrinkage to take place on top of the cast. Lead is used for counter dies. In swaging plates I use a fusible metal for a core to first drive down the plate in the roof or palate, then use a clamp to hold die and core together, while with a horn mallet the outer edge of the plate is made to conform to the alveolar ridge. During this process the plate should be frequently annealed. Two or three counter dies of lead are required to complete the process of making accurately fitting plates.

I will notice briefly some of the materials commonly used as bases for artificial teeth. "Nature's best imitator" celluloid, (formerly *Rose Pearl,*) pyroxline, and coroline, are among the things that were. None of these collodian bases occupy any position, except in the minds of their owners. They are not fit even for temporary work, and no dentist having any regard for his future reputation, will use any of them. Rubber in the hands of competent operators, and especially in the hands of a bungler, can come nearer filling the bill for temporary and cheap sets of teeth, than any other substance now known. But rubber, does not suit all cases, as the "*par excellence*" of bases. Gold will even claim its pre-eminence, it has stood the longest test of time. and experience. *Continuous gum* work, on platinum and iridium, makes strong and the most beautiful work known to the profession.

But when a patient leaves to my judgment, to make the best set of teeth I can, taking everything into consideration, I invariably recommend gold, with rubber attachment, a case well made in this manner will carry with it, character and permanence; such as years cannot tarnish. Let us then emulate all of the higher and nobler attainments of our art; shunning all things which degrade, detract from, or in any degree lower the standard of our profession. But on the contrary let each of us contribute all our power and energy, to exalt and perfect, this, our vocation.

